# Paraneoplastic Arthritis in a Patient With Gastric Cancer: A Case Report

**DOI:** 10.7759/cureus.9692

**Published:** 2020-08-12

**Authors:** Sami Rabah, Donya Bani Hani, Nargis Jilani

**Affiliations:** 1 Internal Medicine, Lincoln Medical Center, New York, USA; 2 Internal Medicine, Lincoln Medical center, New York, USA

**Keywords:** paraneoplastic, arthritis, paraneoplastic arthritis, gastric cancer, rheumatoid arthritis, paraneoplastic syndrome

## Abstract

Paraneoplastic syndromes occur in the presence of a tumor and are known to cause a myriad of systemic manifestations by mechanisms other than direct metastasis. Although considered to be rare, tumors can cause paraneoplastic rheumatological manifestations such as paraneoplastic arthritis. Differentiating between paraneoplastic arthritis and primary rheumatoid arthritis (RA) presents a diagnostic challenge to physicians. Here we describe a case of an 83-year-old male with complaints of painful joint swelling of his hands, elbows, and feet. Subsequent tests ultimately led to the diagnosis of gastric cancer with associated paraneoplastic arthritis. We highlight the physical, laboratory, and imaging findings associated with the diagnosis of paraneoplastic arthritis with emphasis on the differences between this diagnosis and that of RA. Despite the uncommon nature of paraneoplastic arthritis, it remains of paramount importance to be aware of its association with malignancies, aiding in possible earlier diagnosis.

## Introduction

Paraneoplastic arthritis is a rare and yet known rheumatological manifestation of malignancies [1]. Although the diagnosis can be difficult as it mimics rheumatoid arthritis (RA), it has multiple distinguishing features. Here we present a case of an elderly gentleman who presented with new-onset inflammatory polyarthritis and was subsequently diagnosed with gastric cancer.

## Case presentation

An 83-year-old hispanic male with a past medical history of benign prostatic hyperplasia, hyperlipidemia, and pre-diabetes presented for his regular geriatric follow up. On review of systems, the patient reported a loss of appetite with unintentional weight loss and fatigue for the past three months. Upon further questioning, he also reported having painful joint swelling in his hands, elbows, and feet associated with morning stiffness that lasted for 20 min on average. He denied any abdominal pain, nausea, vomiting, or melena. On physical exam, multiple joints were noted to be swollen and tender, including metacarpophalangeal and proximal interphalangeal joints of both hands. Work-up showed a hemoglobin of 9.8 g/dL which was lower than his baseline; C-reactive protein and estimated sedimentation rate were 10.43 mg/dL and 120 mm/h respectively, rheumatoid factor (RF) and anti-cyclic citrullinated peptide (anti-CCP) were negative, X-ray imaging of hands, ankles, and feet showed no erosions or changes suggestive of RA (Table 1).

**Table 1:** Laboratory data on presentation.

Variable	Reference range (adults)	Results
Hemoglobin (g/dL)	12-16	9.8
Mean corpuscular volume (fL)	80-99	86.8
Red cell distribution width (%)	12%-15%	14.7
White cell count (10⁹/L)	4.8-10.8	9.4
Platelets count (10⁹/L)	150-400	544
Alanine aminotransferase (U/L)	13-56	13
Aspartate aminotransferase (U/L)	15-37	21
Alkaline phosphatase (U/L)	40-130	180
C-Reactive protein (mg/dL)	0-0.4	10.43
Estimated sedimentation rate (mm/h)	0-20	120
Rheumatoid factor (IU/mL)	0-13	<10
Cyclic citrullinated peptide antibodies (units)	0-19	14
Uric acid (mg/dL)	3.4-8.8	2.7

**Figure 1:**
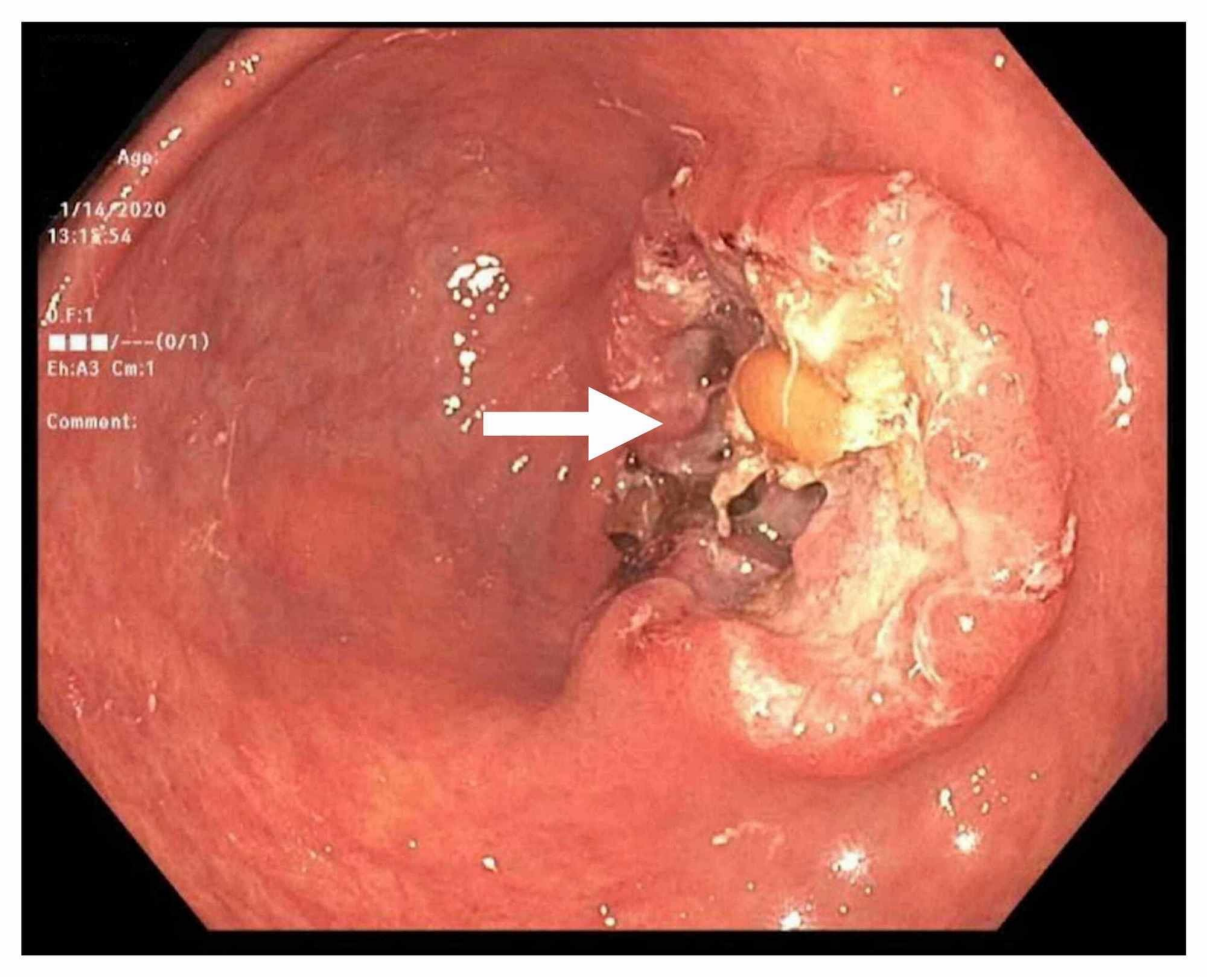
EGD showing a mass in the antrum of the stomach that was later revealed to be an adenocarcinoma. EGD, esophagogastroduodenoscopy

The patient was initially started on NSAIDs for pain relief pending work-up, but he continued to be symptomatic. A referral was subsequently made to rheumatology services as inflammatory arthritis was suspected to be the cause of his symptoms. There he was started on low dose prednisone with some improvement of his symptoms. Given the patient's advanced age along with anemia and weight loss, suspicion was raised to rule out secondary causes of inflammatory arthritis like paraneoplastic syndrome. Extensive work-up including CT imaging for chest, abdomen, and pelvis did not show any findings suggestive of malignancy. Esophagogastroduodenoscopy (EGD) was subsequently done which showed a gastric antral mass (Figure 1). A biopsy confirmed the diagnosis of moderately differentiated adenocarcinoma which was later staged at T3N1mx. Chemotherapy was initiated with surgical options currently being explored.

## Discussion

Paraneoplastic syndromes occur in the presence of a tumor and are known to affect multiple organ systems through biological or autoimmune mechanisms without the tumor directly invading the affected organ [1]. While autoimmune rheumatological disorders are associated with an increased risk of developing certain malignancies, new-onset arthritis in an elderly patient should raise the concern for an occult malignancy as malignant tumors can present with paraneoplastic arthritis and mimic RA [1-2].

Paraneolpastic arthritis is a rare form of arthritis, however, there is currently no published data studying the prevalence or incidence of paraneoplastic arthritis [3]. Paraneoplastic arthritis occurs predominantly in males, with a male-to-female ratio of 1.7:1 [3]. It was found to be associated with several solid and hematological malignancies, with most having lung and breast cancer (mainly adenocarcinoma) followed by leukemia and lymphoma [3-4]. 

The pathogenesis of paraneoplastic arthritis is unknown. But it seems that immunological mechanisms play a role, as one of the proposed mechanisms implicated tumor antigen cross-reactivity with synovium [5]. Our patient presented with features that suggested new-onset RA but had negative serum autoantibodies and his joints did not show any erosions on imaging. However, differentiating early-onset RA from paraneoplastic arthritis remains a diagnostic challenge, as they share common features and symptoms can overlap [4-5].

Both RA and paraneoplastic arthritis are associated with an elevation of acute-phase reactants [4]. Features that are more associated with paraneoplastic arthritis include older age on onset, asymmetry of joint involvement, absence of bone erosions on imaging, and negative rheumatoid factor (RF) [1, 5-6]. Although some case reports suggested that symptoms of paraneoplastic arthritis can antecede the diagnosis of malignancy by 10 months. The results were not statistically significant, as both these conditions can occur simultaneously [3].

When elderly patients present with new-onset arthritis associated with fever, weight loss, or lymphadenopathy, a thorough investigation should be pursued to look for malignancies [3]. Paraneoplastic arthritis should also be suspected when elderly patients present with inflammatory arthritis (with negative imagining and serologies) that is poorly responsive to steroids or disease-modifying antirheumatic drugs (DMARDs) [3]. The course of paraneoplastic synovitis appears to be similar to that of the malignancy, improving with the regression of the associated tumor [3].

## Conclusions

Malignancies can rarely present with paraneoplastic arthritis; a thorough work-up should be pursued when elderly patients present with new-onset arthritis with features suggestive of an underlying malignancy. More studies are needed to elucidate the prevalence, mechanism, and management of paraneoplastic arthritis. 
